# *Pkhd1*^*cyli/cyli*^ mice have altered renal *Pkhd1* mRNA processing and hormonally sensitive liver disease

**DOI:** 10.1007/s00109-023-02351-2

**Published:** 2023-08-16

**Authors:** Chaozhe Yang, Naoe Harafuji, Ljubica Caldovic, Weiying Yu, Ravindra Boddu, Surajit Bhattacharya, Hayk Barseghyan, Heather Gordish-Dressman, Oded Foreman, Zsuzsa Bebok, Eva M. Eicher, Lisa M. Guay-Woodford

**Affiliations:** 1 Center for Translational Research, Children’s National Research Institute, Washington, DC 20010 USA; 2 Center for Genetic Medicine Research, Children’s National Research Institute, Washington, DC 20010 USA; 3 Department of Genomics and Precision Medicine, The George Washington University School of Medicine and Health Sciences, Washington, DC 20037 USA; 4 Division of Nephrology, Department of Medicine, University of Alabama at Birmingham, Birmingham, AL 35294 USA; 5 Department of Pharmacology & Cancer Biology, Duke University School of Medicine, Durham, NC 27710 USA; 6 Genentech USA, Inc, South San Francisco, CA 94080 USA; 7 Cell Developmental and Integrative Biology, University of Alabama at Birmingham, Birmingham, AL 35294 USA; 8 The Jackson Laboratory, Bar Harbor, ME 04609 USA; 9 Children’s Hospital of Philadelphia, Philadelphia, USA

**Keywords:** ARPKD, *Pkhd1*, *Cyli*, Mouse model, Hepato-renal fibrocystic disease

## Abstract

**Abstract:**

Autosomal-recessive polycystic kidney disease (ARPKD; MIM #263200) is a severe, hereditary, hepato-renal fibrocystic disorder that causes early childhood morbidity and mortality. Mutations in the polycystic kidney and hepatic disease 1 (*PKHD1*) gene, which encodes the protein fibrocystin/polyductin complex (FPC), cause all typical forms of ARPKD. Several mouse lines carrying diverse, genetically engineered disruptions in the orthologous *Pkhd1* gene have been generated, but none expresses the classic ARPKD renal phenotype. In the current study, we characterized a spontaneous mouse *Pkhd1* mutation that is transmitted as a recessive trait and causes cysticliver (*cyli*), similar to the hepato-biliary disease in ARPKD, but which is exacerbated by age, sex, and parity. We mapped the mutation to Chromosome 1 and determined that an insertion/deletion mutation causes a frameshift within *Pkhd1* exon 48, which is predicted to result in a premature termination codon (UGA). *Pkhd1*^*cyli/cyli*^ (*cyli*) mice exhibit a severe liver pathology but lack renal disease. Further analysis revealed that several alternatively spliced *Pkhd1* mRNA, all containing exon 48, were expressed in *cyli* kidneys, but in lower abundance than in wild-type kidneys, suggesting that these transcripts escaped from nonsense-mediated decay (NMD). We identified an AAAAAT motif in exon 48 upstream of the *cyli* mutation which could enable ribosomal frameshifting, thus potentially allowing production of sufficient amounts of FPC for renoprotection. This mechanism, expressed in a species-specific fashion, may help explain the disparities in the renal phenotype observed between *Pkhd1* mutant mice and patients with *PKHD1*-related disease.

**Key messages:**

- The *Pkhd1*^*cyli/cyli*^ mouse expresses cystic liver disease, but no kidney phenotype.
- *Pkhd1* mRNA expression is decreased in *cyli* liver and kidneys compared to wild-type.
- Ribosomal frameshifting may be responsible for *Pkhd1* mRNA escape from NMD.
- *Pkhd1* mRNA escape from NMD could contribute to the absent kidney phenotype.

**Supplementary Information:**

The online version contains supplementary material available at 10.1007/s00109-023-02351-2.

## Introduction

Autosomal-recessive polycystic kidney disease (ARPKD; MIM #263200) is a hereditary hepato-renal fibrocystic disorder with an estimated incidence of 1 in 26,500 live births [1]. In most patients, ARPKD is characterized by the cystic dilatation of renal collecting ducts, causing progressive renal insufficiency and ultimately end-stage kidney disease [2, 3]. In the liver, the ductal plate malformation gives rise to congenital hepatic fibrosis, which often leads to portal hypertension [2, 3]. Virtually, all cases of typical ARPKD are caused by mutations within the polycystic kidney and hepatic disease 1 (*PKHD1*) gene located on chromosome 6p21.1 [4–6]. The full-length *PKHD1* transcript is composed of 67 exons with the longest open reading frame (ORF) encoding a 4074 amino acid protein called fibrocystin or fibrocystin/polyductin complex (FPC) [4, 5]. Despite the passage of two decades since the identification of *PKHD1* as the genetic determinant of ARPKD, the function of FPC remains poorly understood beyond its suggested roles in E3 ubiquitin ligase complex and SRC/STAT3 signaling [7, 8].

Several orthologous mouse models of ARPKD have been described (Table 1), primarily generated through random mutagenesis or targeted genetic engineering of the *Pkhd1* gene [9–16]. Virtually, all mutant *Pkhd1* mice exhibit a liver phenotype resembling human disease, but renal cystic disease is either absent or very mild and slowly progressive [3]. The mouse *Pkhd1* locus, located on Chromosome 1qA3-4, consists of 67 non-overlapping exons encoding a protein of 4059 amino acids [17]. Human and mouse FPC share 87% overall identity across domains encompassing a predicted N-terminal signal peptide, multiple immunoglobulin-like plexin domains, multiple parallel β-helix 1 repeats, and a single transmembrane domain. In contrast, the short C-terminal cytoplasmic domains of mouse and human FPC are only 40% identical [17, 18].

**Table 1:** ARPKD orthologous mouse model phenotypes

Strain	Background	Kidney	Liver	Pancreas	References
*Pkhd1*^*lacZ*^	129S; B6	PT dilatation	DPM	Cystic	Williams et al. [14]
*Pkhd1*^*LSL(−),Pk(+)*^	BALB/cJ, C57BL/6	PT dilatation	DPM	Cystic	Bakeberg et al. [19]
*Pkhd1*^*del2*^	BALB/cJ, C57BL/6	PT dilatation	DPM	Cystic	Woollard et al. [10]
*Pkhd1*^*del3–4*^	129S; B6	TAL/CD dilatation	DPM	Cystic	Garcia-Gonzalez et al. [11]
*Pkhd1*^*del4*^	129S; B6	None	DPM	Cystic	Gallagher et al. [12]
*Pkhd1*^*e15GFPdel16*^	C57BL/6	PT/MCD dilatation	DPM	None	Kim et al. [13]
*Pkhd1*^*del40*^	C57BL/6	None	DPM	----	Moser et al. [9]
*Pkhd1*^*cyli*^	D.B/11Ei	None	DPM	None	Current study
*Pkhd1*^*del67*^	C57BL/6	None	None	None	Outeda et al. [16]

*PT* proximal tubule, *TAL* thick ascending limb of Henle, *MCD* medullary collecting duct, *CD* collecting duct, *DPM* ductal plate malformation

In mice, in addition to the full-length *Pkhd1* mRNA, multiple alternatively spliced *Pkhd1* transcripts have been reported [17–19], whereas in humans, *PKHD1* appears to be less transcriptionally complex [20]. Our previous work identified 22 alternative *Pkhd1* transcripts in wild-type/normal (WT) mice [17, 18]. This is consistent with the presence of several intronic and exonic splicing enhancers (ISE, ESE) that are likely to play roles in the canonical as well as alternative splicing of the *Pkhd1* mRNA [18]. In comparison, human *PKHD1* is much less transcriptionally complex, with only 2 validated alternatively spliced isoforms (https://www.ncbi.nlm.nih.gov/gene/5314).

Here, we report the identification of a new, spontaneously occurring *Pkhd1* mutation, that causes cysticliver (*cyli*), similar to the hepato-biliary disease in ARPKD. We describe the liver phenotype of *cyli* mice, mapping of the *cyli* mutation to the *Pkhd1* gene, and comparative studies of *Pkhd1* transcript profiles and abundance in WT and *cyli* mice. The phenotype of *Pkhd1*^*cyli/cyli*^ (*cyli*) mice is similar to other *Pkhd1* mutant models with liver cystic disease and no/minimal renal involvement. The insertion/deletion (indel) mutation in exon 48 results in a frameshift and a predicted premature termination codon (PTC) (UGA). In characterizing the *Pkhd1*-derived transcripts*,* we noted multiple differences in WT and *cyli* kidney-derived mRNAs, but observed exon 48 containing transcripts from both WT and mutant kidneys.

## Materials and methods

### Mice

All mouse experiments were approved by the *Institutional Animal Care and Use Committees* at The Jackson Laboratory, University of Alabama at Birmingham (UAB) and Children’s National Research Institute (CNRI). The study was conducted in accordance with relevant guidelines and regulations in the Guide for the Care and Use of Laboratory Animals of the National Institutes of Health. The Jackson Laboratory, UAB, and the Comparative Medicine Unit (previously named Research Animal Facility) at Children’s National Medical Center are fully accredited by the AAALAC.

The D.B/11Ei congenic inbred mouse strain was generated by introgression of a segment of distal Chromosome 4 from C57BL/6J (B6) onto the DBA/2J background. The first affected mouse noted was a 5-month-old (22-week-old) D.B/11Ei female breeder, generation N11F13. This female had successfully raised two litters, was pregnant with a third litter, and appeared sick. Further investigation revealed a hard, distended abdomen containing an enlarged liver with yellow, fluid-filled cysts. Liver disease was not evident in the male breeder. Offspring from this pair and closely related mice were monitored for signs of liver disease. These offspring were used to establish the D.B/11Ei strain carrying the recessive *cyli* allele and the results reported here are derived from the original breeding pair.

The D.B/11Ei-*cyli* strain used in this study was first transferred to UAB and subsequently to Children’s National Research Institute (CNRI). Because affected mice survive into adulthood and are capable of reproducing, the *cyli* mutation can be maintained using homozygous breeding of the D.B/11Ei-*cyli* strain.

### Locus mapping, gene identification, and mutation sequencing

A standard backcross mating scheme was used to identify the chromosomal location of the *cyli* gene [21–23]. F_1_ females, produced by mating B6 to D.B/11Ei-*cyli* mice, were backcrossed to D.B/11Ei-*cyli* males. The backcross offspring (*n* = 221) was evaluated at 22 weeks of age for the presence of cystic liver disease. An initial genetic variant mapping approach and subsequent fine-mapping studies were performed using MIT microsatellite markers [24]. The introgressed B6 segment from Chromosome 4 was excluded as a candidate disease interval. A disease-associated interval that contained the *Pkhd1* locus was identified on Chromosome 1. DNA sequencing of *Pkhd1* exons and flanking intronic sequences were amplified by PCR and the amplicons sequenced in both directions using primer sets (Supplementary Table 1) designed based on the published *Pkhd1* gene sequence [17].

### Mouse genotyping

DNA for genotyping was isolated from biopsied tail tissue. Tissue was lysed at 55 °C in Cell Lysis Solution (Qiagen, # 158116) containing Proteinase K (Qiagen, # 19133), followed by protein precipitation with the Protein Precipitation Solution (Qiagen, # 158126) for 10 min at −20 °C. Sample was then centrifuged at 16,000 × g for 10 min at 4 °C. Genomic DNA was precipitated from the supernatant with ethanol, pelleted by centrifugation at 16,000 × g for 5 min at 4 °C, air dried, and resuspended in water. PCR-based genotyping was performed using primers 5′-TGG CTA TAC TGT GAA GAC CAG GCA-3′ (forward) and 5′-AAG CTT GGG CCT ATC TGA ATG GCA-3′ (reverse) and the following conditions: 15 min at 95 °C initial denaturation, followed by 35 cycles of 45-s denaturation at 94 °C, 45-s annealing at 52 °C, and 1-min extension at 72 °C, with a 10-min final extension at 72 °C. PCR products were digested with *Bsa*I, and the products were resolved by agarose gel electrophoresis. Bands of 126 bp and 359 bp were diagnostic of the WT gene and a 484 bp band identified the *cyli* mutant allele.

### Tissue histology and morphometric analysis

Kidneys and livers were harvested from male and female WT and *cyli* mice, paraffin embedded, sectioned, deparaffinized, rehydrated, and stained with hematoxylin and eosin (H&E) according to standard protocols [25–28]. Stained tissue sections were examined by light microscopy using an Olympus CX41 microscope equipped with a Leica DX320 color camera and Leica software. Histomorphometry was performed on blinded experimental specimens by a veterinary pathologist according to previously described protocols [29]. Images were collected using a Nikon E600 microscope equipped with a SPOT Insight digital camera (Diagnostic Instruments) and analyzed using Image-Pro Plus v6.2 image analysis software (Media Cybernetics Inc). Cyst and tissue areas were quantified by converting images to grayscale and thresholding them to produce a black image on a white background. Cysts were represented as white objects within the image. Cystic and total (including cysts) areas were determined automatically using the count/size and macro functions of Image-Pro Plus. The results were expressed as % of cyst area relative to total area.

For the initial evaluation of disease course in D.B/11Ei-*cyli* line, mice were weaned at 3 weeks of age and assigned to a specific age group for timed tissue harvesting. To investigate liver disease progression in females as a function of parity, 3 sib-mated pairs were assigned for tissue harvesting after the birth of their first litter, and 3 sib-mated pairs were assigned for tissue harvesting after the birth of their second litter.

### Reverse transcription (RT)–PCR and quantitative (q)RT-PCR

Total RNA samples from kidneys and livers harvested from 4.5- and 7-week-old WT and *cyli* mice were prepared using RNeasy Mini kit (Qiagen, # 74104), treated with RQ1 RNase-Free DNase (Promega, # M6101), and then re-purified using the RNeasy Mini kit. For RT-PCR, RNA samples were reverse transcribed using SuperScript III First-Strand Synthesis SuperMix (Thermo Fisher Scientific, # 18080400) and oligo dT primers. RT-PCR was performed to compare *Pkhd1* transcripts in WT and *cyli* kidney and liver tissue using primers specific for *Pkhd1* exons 1 (forward: 5′-CAT TTG AGG CAC AAG GCT GAC ACA-3′) and 67 (reverse: 5′-CTG AGG TCT GGG CGT AAC AG-3′) sequences. Relative *Pkhd1* transcript abundance in WT vs. *cyli* kidneys was determined by quantitative real-time PCR performed on a QuantStudio 7 Flex Real-Time PCR System (Thermo Fisher Scientific) using the default program. PCR was performed with cDNA templates using Power SYBR Green PCR Master Mix (Thermo Fisher Scientific, # 4368706) and primers specific for sequences of *Pkhd1* exons 2–5 (forward: 5′-ATG ATG CTT GCC TGG CTG GTC-3′; reverse: 5′-TAT GGC CCT GCA TCT GCT TCT GAT-3′), *Pkhd1* exons 5–6 (forward: 5′-GTC TCT TCC ATC AGA AGC AGA TGC-3′; reverse: 5′-GGG TAA ACT TGA TAT AAA ACA GG-3′), *Pkhd1* exons 37 (forward: 5′-CAT GGA TCC AGG ACC CAT TG-3′; reverse: 5′-AGG GAA AGA AGG GAG TGG AA-3′), *Pkhd1* exons 48–49 (forward: 5′-TGG CTA TAC TGT GAA GAC CA-3′; reverse: 5′-GAT CCA AGA GCA GAG CCA TC-3′), *Pkhd1* exons 61–62 (forward: 5′-TCA CTC TTG AGA TGC CTG GC-3′; reverse: 5′-AGG TTC CCA GTT ATT AAA CTA C-3′), and *Pkhd1* exons 66–67 (forward: 5′-CCA GAA GAC ATA TCT GAA TCC CAG GC-3′; reverse: 5′-AGC AAG AGA TCC TGG AAC ACA GGT-3′). *Peptidylprolyl Isomerase A* (*Ppia*) was used for normalization (forward: 5′-AGC ACT GGA GAG AAA GGA TT-3′; reverse: 5′-ATT ATG GCG TGT AAA GTC ACC A-3′) [30]. Results were analyzed using QuantStudio Real-Time PCR Software and the ΔΔCt method [31]. The results were graphed with GraphPad Prism version 9.1.2 for Windows, GraphPad Software, San Diego, CA, USA, www.graphpad.com. Expression levels in *cyli* mice were normalized to their respective WT values. Comparison of expression levels in *cyli* mice was compared to a constant value of 1.0 using a two-sided Wilcoxon sign rank test.

### Immunoblotting

Kidneys were collected from 2-week-old WT and *cyli* mice and immediately snap frozen in liquid nitrogen. Kidneys were homogenized on ice for 20 s in 1mL ice-cold RIPA buffer (Sigma-Aldrich, # R0278) containing proteinase inhibitors (Protease Inhibitor Cocktail Mini-Tablet EDTA-free, Bimake, # B14012). Homogenates were centrifuged for 10 min at 15,000 × g at 4 °C. Protein concentration in supernatants was determined using BCA protein assay kit (Thermo Scientific, # 23227). Twenty micrograms of total protein was mixed with NuPAGE LDS sample buffer (Life Technologies, # NP0007) containing sample reducing agent (Life Technologies, # NP0009). Samples were heated at 100 °C for 10 min prior to electrophoresis through a Novex NuPAGE 4–12% Bis-Tris gel (Life Technologies, # NP0335BOX) in MES SDS running buffer (Life Technologies, # NP0002) for 30 min at 200 V. Proteins were transferred to a polyvinylidene fluoride membrane using Bio-Rad Trans-Blot Turbo Transfer System. The membrane was incubated with rat anti-mouse FPC monoclonal antibody PD1E1, which was obtained from the Baltimore PKD Center [16], in 1× PBS plus 0.1% Tween-20 (PBST) with 5% bovine serum albumin overnight at 4 °C. The membrane was washed 3 times 10 min with 1× PBST, then incubated with goat anti-rat secondary antibody (Thermo Fisher Scientific, # 31475) 1:5000 dilution in 1× PBST with 5% non-fat dry milk for 1 h at room temperature, followed by 3 washes with 1× PBST. Immunoreactive bands were detected using SuperSignal West Dura chemiluminescent substrate (Thermo Fisher Scientific, # 34076) and imaged using Bio-Rad ChemiDoc MP Imaging System. Densitometry of FPC bands was performed with Bio-Rad Image Lab Software 6.1 and normalized to GAPDH. The relative intensity was calculated with WT as 1.00. The result was graphed with GraphPad Prism version 9.1.2 for Windows. Two-tailed Student’s *t*-test was used for statistical analysis.

### Targeted long-read cDNA sequencing

Approximately 1μg of PCR-amplified kidney cDNA from 7-week-old WT and *cyli* mice was used to perform nanopore-based long-read sequencing. SQK-LSK109 Amplicon sequencing kit [Oxford Nanopore Technologies (ONT)] was used for library preparation. Briefly, DNA repair and end-prep were accomplished with NEBNext FFPE DNA Repair and Ultra II End-Prep reaction and enzyme mixes (New England Biolabs). AMPure XP beads were then used to bind DNA and prepared for adapter ligation with NEBNext Quick t4 DNA ligase and adapter mix (ONT). Long fragment buffer was used to select for reads greater than 3 kb in size. The resultant DNA was primed and loaded onto the ONT Flongle flowcell. Each sample was run for 24 h, generating approximately 300 Mbp of raw reads. Quality estimation and visualization of the raw fastq files were performed using *NanoPlot* and *nanoQC* functions of the *nanoPack* suite of tools [32]. The fastq reads were then mapped to the mouse reference genome (GRCm39) (https://www.ncbi.nlm.nih.gov/assembly/GCF_000001635.27/) using minimap2 [33]. To estimate the read coverage per exon, the aligned bam files were first sorted and indexed using *samtools* [34], followed by read coverage estimation per kilobase using bamCoverage [35]. The output bed files were then read and merged with sample metadata using *LoadTrackFile* function of *ggcoverage* [36], followed by mapping to the 67 exon locations using a custom R script. Integrative Genomics Viewer (IGV) and Adobe Illustrator were used to visualize *Pkhd1* splicing patterns in WT and *cyli* kidneys after exclusion of reads shorter than 1 kb.

## Results

### Discovery of the *cyli* mutant mouse

**Fig. 1:**
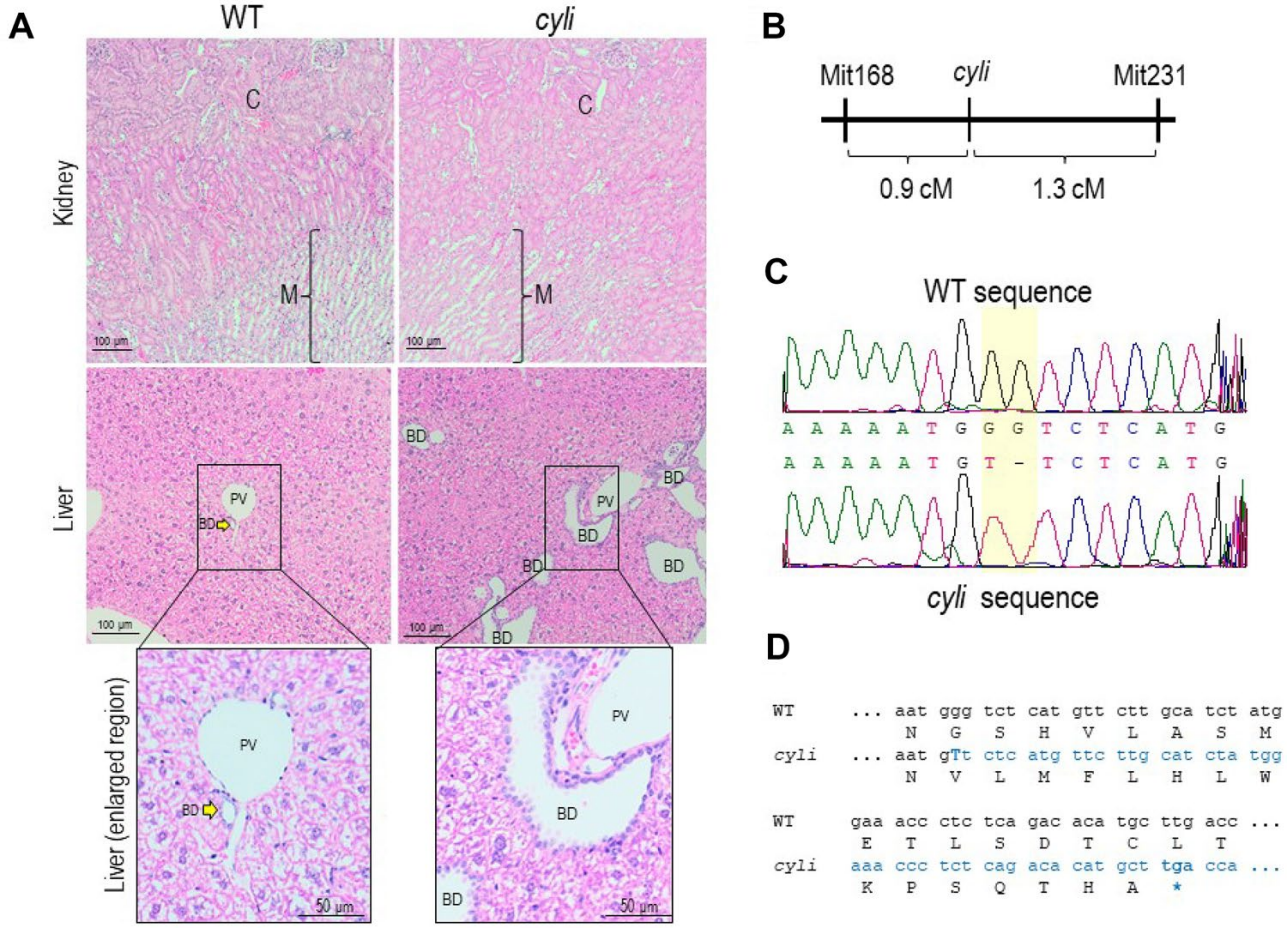
Initial characterization of the *cyli* mouse model. **A** Kidney (top) and liver (middle and bottom) tissue sections stained with hematoxylin and eosin (H&E) from 8-week-old WT and *cyli* mice. Bottom panels are higher magnification views of the boxed areas in middle panels (BD, bile duct; C, cortex; M, medulla; PV, portal vein). **B** Schematic illustrating the position of the *cyli* mutation (affecting *Pkhd1*) on Chromosome 1 between the genetic markers *D1Mit168* and *D1Mit231* (genetic distances are in centiMorgan (cM) units). **C** Sequence comparison of *Pkhd1* from WT and *cyli* mice (NM_153179.3: c.7588_7589delGGinsT (p.G2530VfsTer15). **D** Comparison of WT and *cyli* reading frames. The *cyli* mutation leads to the formation of a PTC that is predicted to cause premature protein truncation

Investigation of a sick D.B/11Ei female mouse revealed an enlarged liver containing multiple fluid-filled cysts. Subsequently, we determined that this disorder was transmitted as a recessive trait. Homozygous *cyli* mutant mice developed cystic liver disease around 4 weeks of age. Histopathological analysis demonstrated biliary dysgenesis characterized by ductal plate malformation phenocopying the liver lesion characteristic of human ARPKD, with portal tracts of affected livers exhibiting multiple irregularly shaped and variably dilated bile ducts lined with hyperplastic epithelium (Fig. 1A). However, there was no histopathologic evidence of renal cystic disease in *cyli* mutants examined between 8 and 11 weeks of age (Fig. 1A).

### Gene identification and mutation analysis

The disease-causing locus was positioned to Chromosome 1 between markers *D1Mit168* and *D1Mit231* (*D1Mit168* - 0.9 cM – *cyli* - 1.3 cM – *D1Mit231*), the region containing the *Pkhd1* gene (Fig. 1B). Sequence analysis of *cyli* mice identified an indel mutation NM_153179.3:c.7588_7589delGGinsT (p.G2530VfsTer15) in *Pkhd1* exon 48 (Fig. 1C) leading to a frameshift and predicted PTC within exon 48, 44 bp downstream of the T insertion (Fig. 1D).

### Progressive liver disease modulated by sex, age, and parity

**Fig. 2:**
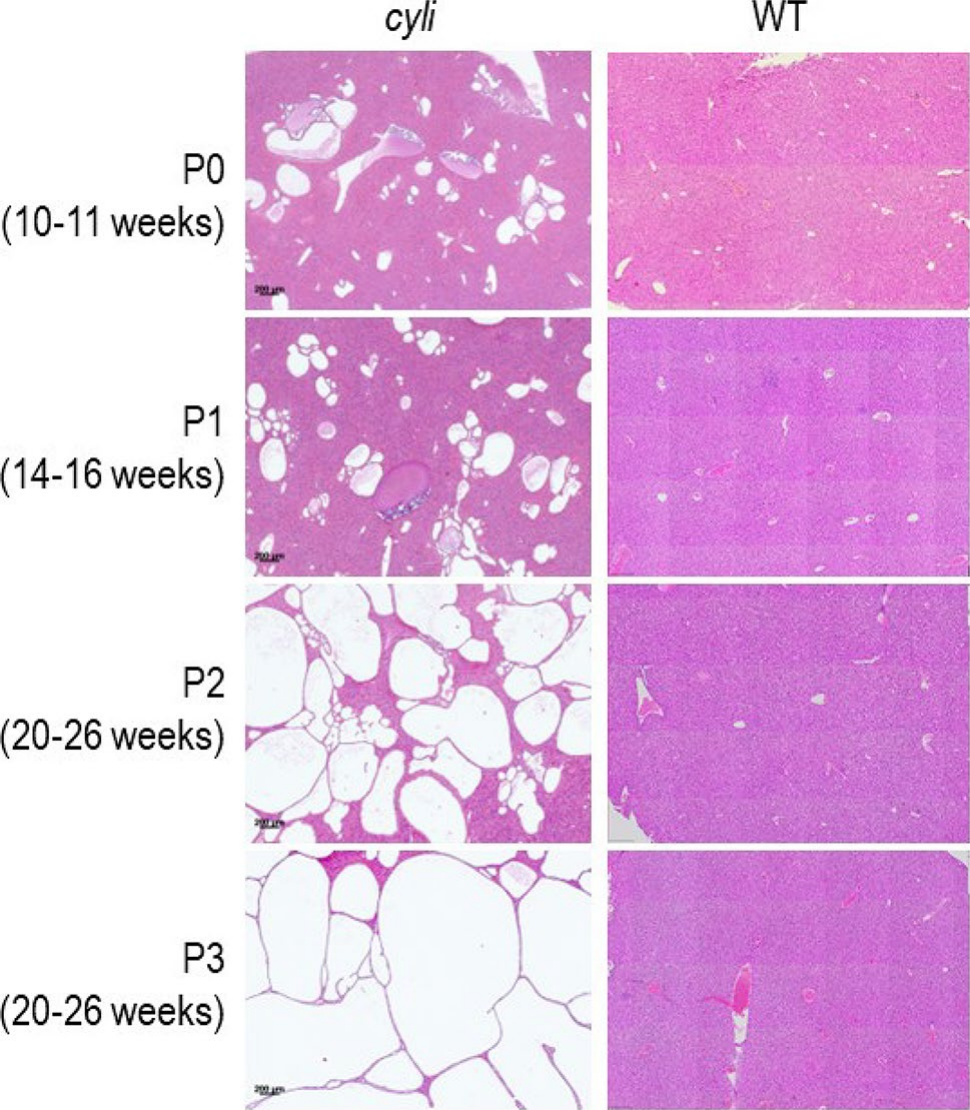
Progressive cystic liver phenotype in *cyli* mutant female mice. Representative hematoxylin and eosin (H&E) stained liver sections from female *cyli* mice between 4 and 26 weeks of age demonstrate progressive liver cyst formation and severely reduced typical liver parenchyma. P0 (10–11 weeks), P1 (14–16 weeks), P2 (20–26 weeks), and P3 (20–26 weeks) denote numbers of litters produced and the age of *cyli* females (left). The significantly larger cysts in P3, 20–26 weeks old mice (bottom left), suggest that in addition to age, parity also contributes to the severity of the disorder. Age-matched WT liver sections are shown on the right. Scale bar = 200 µm

Morphometric analysis of liver sections from female and male *cyli* mice at multiple time points revealed an age-associated increase in the severity of liver disease. Younger *cyli* mice (less than 11 weeks old) displayed a liver phenotype characterized by dilated bile ducts radiating from the portal region into the parenchyma (Fig. 2 and Table 2). By week 14, mice displayed coalescing cysts progressively replacing areas of the liver parenchyma. Beyond 20 weeks of age, female mice tended to exhibit more extensive cystic lesions than male mice of comparable age (Table 2). Comparing liver cyst formation at 20–26 weeks of age between mice with two or three pregnancies (P2, P3) suggested that in addition to age and sex, parity may also contribute to the severity of the cystic liver disorder (Fig 2, left, bottom two images and Table 2). The study did not extend phenotypic examination of mice beyond 26 weeks of age or 3 pregnancies. A detailed longitudinal comparison of age-matched nulliparous female mice (separated from male littermates prior to the first pregnancy) to female mice with specific number of pregnancies was beyond the scope of the current project.

**Table 2:** Progressive cystic liver disease in *cyli* mice

Age (weeks)	Sex	*N*	Phenotype		Parity
			Histomorphology	Cystic area (% of total)	
1	M	4	Hyperplastic bile ducts	N/A	--
	F	3			0
4	M	3	Dilated bile ducts	N/A	--
	F	5			0
6	M	3	Hyperplastic, dilated bile ducts	N/A	--
	F	2			0
10–11	M	1	Coalescing cystic bile ducts	25	--
	F_n_	2		15–20	0
14–16	M	4	Coalescing cystic bile ducts	15–35	--
	F_p_	3		20–35	1
20–26	M	7	Coalescing cystic bile ducts	30–70	--
	F_p_	7		30–80	2-3

*F*_*n*_ female nulliparous, *F*_*p*_ female parous

### Differential *Pkhd1* transcript profiles in WT vs. *cyli* mice

**Fig. 3:**
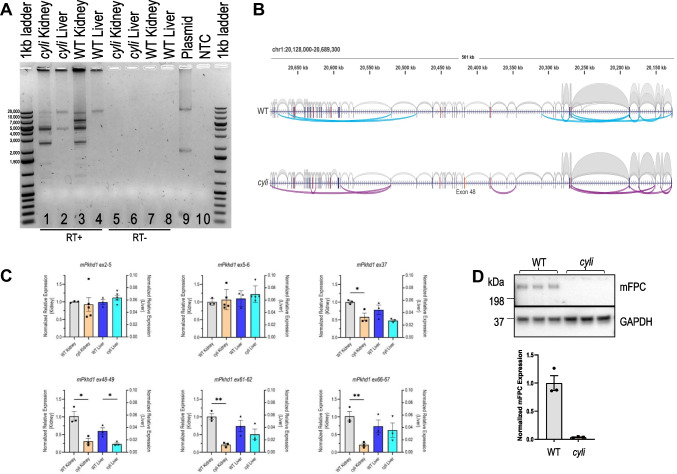
*Pkhd1* expression in WT and *cyli* mice. **A** *Pkhd1* transcript (mRNA) profiles amplified from total kidney and liver RNAs of 7-week-old WT and *cyli* male mice. PCR products (amplicons) were generated from oligo dT primed template cDNA using primers specific for *Pkhd1* exons 1 and 67. In lane 9 (Plasmid), the band at 2000 bp represents either a non-specific or plasmid recombination-derived amplification product. **B** Visualization of *Pkhd1* mRNA splicing patterns in the kidneys of 7-week-old WT and *cyli* mice, respectively. Gray – forward and reverse RNA-seq reads spanning adjacent/consecutive *Pkhd1* exons. Cyan – forward and reverse RNA-seq reads spanning alternatively spliced *Pkhd1* exons in WT kidneys. Purple – forward and reverse RNA-seq reads spanning alternatively spliced *Pkhd1* exons in *cyli* kidneys. Arrows indicate the direction of transcription. Exons 10, 20, 30, 40, 50, and 60 are indicated in red. Exon 48 is shown in orange. **C** Relative expression of *Pkhd1* mRNA containing exons 2–5, 5–6, 37, 48–49, 61–62, and 66–67 in the kidneys and liver of 4.5-week-old WT and *cyli* female mice. Data were normalized to *Ppia* mRNA; expression of *Pkhd1* mRNA in WT mouse kidneys (left y-axis) and liver (right y-axis) was set as 1.00. Data were expressed as mean ± S.E; *n*=3 or 5 per group. **D** Immunoblot of FPC protein in kidney lysates from 2-week-old WT and *cyli* male mice using a rat monoclonal primary antibody specific for *Pkhd1* exon 67-encoded amino acid sequences [16]. Data were expressed as mean ± SEM; *n*=3 mice per group

We previously demonstrated that WT *Pkhd1* mRNA is subject to extensive alternative splicing in the kidney but not in the liver and that the longer transcripts typically include exon 48, where the *cyli* indel mutation occurs [18]. To evaluate whether the *cyli* mutation altered the alternative splicing products, we compared *Pkhd1* transcripts from WT and *cyli* kidneys as well as the corresponding livers (Fig. 3A). The *Pkhd1* cDNA was amplified using primers specific for *Pkhd1* exons 1 and 67. Consistent with previously published findings [18], we identified four major products of 12, 6.5, 4.5, and 2.5 kb, as well as some additional minor bands, representing the full-length and major alternatively spliced *Pkhd1* mRNAs from WT kidneys (Fig. 3A, lane 3) [18]. In WT liver, we observed a 12 kb product (Fig. 3A, lane 4) while in *cyli* liver, we identified a 12 kb and a 4.5 kb band (Fig. 3A, lane 2). As in previous studies, we were able to reproducibly amplify a 4.5 kb product from WT liver RNA [18], but the 4.5 kb band was not reproducibly detectable in liver RNA from *cyli* mice. This variability in detection may reflect the very low transcript abundance in total liver RNA as *Pkhd1* is expressed only in cholangiocytes which represent approximately 5% of the total liver cellular mass [37].

Next, we used ONT long-read sequencing technology to examine the splicing patterns of *Pkhd1* transcripts in WT and *cyli* kidneys. Abundance of *Pkhd1* transcripts in the liver was too low for ONT sequencing. Distribution of read lengths from sequencing WT *Pkhd1* transcripts showed maxima at approximately 2.5, 4.5, 6.5, and 12 kb (Supplementary Fig. S1) which corresponded to the sizes of WT *Pkhd1* cDNA amplification products (Fig. 3A). Distribution of read lengths from sequencing *cyli Pkhd1* transcripts had maxima at approximately 2.5 and 4.5 kb, and included long reads that corresponded to full-length *Pkhd1* transcripts (Supplementary Fig. S1). Again, these data correspond to sizes of *cyli Pkhd1* cDNA amplification products (Fig. 3A). Visualization of *Pkhd1* splicing patterns showed the presence of transcripts containing all 67 exons in both WT and *cyli* kidneys (Fig. 3B). Splicing of *Pkhd1* exons 6 and 37 has been observed previously in the kidneys from WT mice [18]. Other alternative slicing events in WT *Pkhd1* transcripts are novel (Fig. 3B) and underscore the complex pattern of the *Pkhd1* splicing in mouse kidneys. While patterns of alternatively spliced *Pkhd1* exons differed in WT and *cyli* kidneys, exon 48 was present in the *cyli Pkhd1* transcripts, indicating that full-length FPC could be translated in mutant kidneys (Fig. 3B).

We then used qRT-PCR and primer-pairs specific for junctions spanning exons 2–5, 5–6, 37, 48–49, 61–62, and 66–67 to examine differential amplicon profiles and confirm the presence of exon 48 in the population of *cyli* derived mRNAs. We observed significantly lower levels of amplification products in both WT and *cyli* livers compared to kidneys for all targeted amplicons (Fig. 3C, right y-axis for the kidney and left y-axis for the liver). The abundance of amplicons spanning exon 37, which is alternatively spliced in WT kidneys [18], was lower in *cyli* than in the WT kidneys and livers (Fig. 3C). The abundance of amplicons spanning exons 48–49, 61–62, and 66–67 was lower in *cyli* than in the WT kidneys (Fig. 3C). Unlike in kidneys, the differences in abundance of amplicons spanning exons 48–49, 61–62, and 66–67 were not statistically significant in the liver samples, perhaps because *Pkhd1* transcripts were in very low abundance due to their expression in cholangiocytes. Lower abundance of amplicons spanning exons 48, 61–62, and 66–67 in *cyli* than in the WT kidneys and livers suggests that a fraction of *Pkhd1* transcripts in *cyli* kidneys escape NMD.

In addition, we investigated expression of FPC in WT and *cyli* kidneys and livers using an antibody generated against an exon 67–encoded epitope [16]. We detected full-length FPC in WT kidney protein extracts (Fig. 3D). In contrast, full-length FPC was below the detection limit in extracts prepared from *cyli* kidneys (Fig. 3D). Full-length FPC was not detected in lysates from either WT or *cyli* livers (Supplementary Fig. S2). Again, this is likely due to the low abundance of FPC-expressing cholangiocytes in the liver compared to hepatocytes, which do not express FPC. The lower (20–171 kD) molecular weight bands observed in lysates from both WT and mutant livers at the same intensity likely represent non-specific cross-reacting proteins (Supplementary Fig. S2).

## Discussion

The mouse *Pkhd1*^*cyli*^ mutation arose spontaneously due to a de novo indel mutation, NM_153179.3:c.7588_7589delGGinsT, in exon 48 of the *Pkhd1* gene, which causes a frameshift and results in a predicted PTC. Similar to other *Pkhd1* mutant models, *cyli* mice express a hepato-biliary phenotype that progresses with age. Our longitudinal analysis revealed that female sex and pregnancy exacerbate liver disease severity. This sexual dimorphism in *cyli*-related liver disease severity has not previously been reported for human *PKHD1*-related disease or in other genetically engineered *Pkhd1* mouse mutants, possibly due to the paucity of longitudinal studies in both human patients and previously reported mouse models. Our data suggests that, at least in *cyli* mutants, hepato-biliary disease progression may be hormonally modulated, as has been observed in human ADPKD and mouse *Pkd1* models [38]. Further studies will be required to explore this intriguing pathogenic mechanism in both human patients and the multiple *Pkhd1* mouse models.

The most striking feature of all mouse *Pkhd1* models described to date is the absent or minimal renal cystic phenotype [9–14, 16, 19]. In contrast, truncating human *PKHD1* mutations typically cause severe renal cystic disease during fetal life [3]. In the current study, we show that *cyli* mice with an indel mutation in exon 48 do not express renal cystic disease when aged to at least 26 weeks. However, patients with frameshift mutations involving *PKHD1* exon 48 express the classic ARPKD phenotype with severe renal cystic disease expressed in infancy (http://www.humgen.rwth-aachen.de) [39, 40].

The mouse *Pkhd1* gene, unlike its human orthologue, is transcriptionally complex in the WT kidney with a number of alternatively spliced isoforms. In *cyli* mutant kidneys, the majority of the transcripts were 2.5 and 4.5 kb. In our previous study, we demonstrated that these lower molecular weight amplicons contain isoforms that result from exon-skipping events, but maintain the FPC open reading frame (ORF) [18]. For example, the 2.5 kb amplicon contains an isoform resulting exon 6–61 splicing. Similarly, among the transcripts in the 4.5 kb amplicon, some of the products originate from exon 4–49 splicing and exon 6 can be spliced to exons 51, 52, or 53 as well (Supplementary Fig. S3). The long-read sequencing of *Pkhd1* transcripts from WT kidneys detected previously observed splicing of exons 6 and 37 [18] in both WT and *cyli* kidneys. Therefore, a diverse set of alternative *Pkhd1* splice variants observed in the *cyli* kidneys could encode multiple novel isoforms of FPC with sufficient residual function to attenuate cystogenesis in mutant kidneys. In contrast, *Pkhd1* in the WT liver has minimal transcriptional complexity and the full-length *Pkhd1* transcript is predominant [4, 18]. Therefore, the *cyli* liver with limited alternatively spliced *Pkhd1* mRNAs would not have functional redundancy, resulting in the development of the hepato-biliary lesion.

Since the transcripts from the *cyli* kidney did not fully exclude exon 48, we sought mechanisms that could explain this apparent evasion from NMD. Ribosomal frameshifting, which controls polyamine biosynthesis in mammalian cells [41], is one such mechanism. Indeed, sequence analysis of exon 48 revealed an AAAAAT motif upstream of the *cyli* indel. This motif has been shown to promote ribosome shifting on the reading frame by +1 or −1 [42]. For example, if ribosomes shift −1, instead of reading GGG, coding for Gly2530, the ribosome would read TGT, coding for Cys, and proceed with in-frame translation of the FPC sequence.

At the protein level, immunoblotting detected only full-length FPC in WT kidneys. In contrast, bands corresponding to full-length FPC were not observed in immunoblots of *cyli* mouse kidney protein extracts. This finding is consistent with the reduced levels of *Pkhd1* gene expression demonstrated by qRT-PCR and our previous analysis [18] and suggests the possibility that variant transcripts and/or ribosomal frameshifting give rise to very low abundance isoforms of functional FPC, which are capable of abrogating cystogenesis in this model.

While the basis for *PKHD1/Pkhd1* species–specific differences in renal disease expression is not understood, differences in the phenotypic severity between orthologous human disease and mouse models are not uncommon. For example, the *mdx* mouse model of Duchenne muscular dystrophy (DMD) has a mild phenotype compared to human DMD patients [43]. Although *mdx* mice exhibit muscle histopathology, elevated plasma pyruvate kinase and creatine kinase levels, and muscle weakness similar to DMD patients, *mdx* mice are viable and fertile [43], whereas human DMD is a fatal degenerative muscle disorder [44]. Similarly, mouse models of cystic fibrosis (CF) also do not fully recapitulate the human disease (for review: [45]). The numerous mouse CF models exhibit defects in fluid secretion and develop severe intestinal and mild pancreatic disease, but fail to develop the signature lung infections that are the major cause of mortality in human CF [46–49]. The basis for this species-specific difference in lung phenotype appears to involve anatomical differences between the mouse and human airways [50] as well as pH differences in the airway surface liquid in human CF patients vs. mouse models [40].

We speculate that the minimal renal cystic disease in mouse *Pkhd1* models reflects a combination of mechanisms. Based on our data in this specific model, ribosomal frameshifting may allow alternatively spliced mutant transcripts to escape NMD. More broadly, variations in molecular interactions which may be dictated by genetic background rather than *Pkhd1* genotype per se might act to modulate the degree to which a renal cystic phenotype is expressed in mouse models [11, 51, 52]. Finally, we speculate that FPC functions differently in species-specific molecular pathways. Informatic analyses indicate that the short C-terminal cytoplasmic domains of mouse and human FPC are only 40% identical [17, 18], raising the possibility that these protein domains have different intracellular binding partners and participate in different molecular pathways. To this point, we note that mice homozygous for the *Pkhd1* exon 67 deletion (*Pkhd1*^*del67*^), which removes most of the FPC carboxy terminus domain, have no renal or biliary phenotype [16] and mice lacking virtually the entire *Pkhd1* locus (exons 3 through 67) express the hepato-biliary lesion, but only a minor renal phenotype in older mice [19, 53]. In contrast, the corresponding defects in human patients, which involvines loss of a functional carboxy terminus [39, 54] or large intragenic deletions [55, 56] are associated with both the hepato-biliary lesion and severe renal cystic disease.

As noted above for CF, identifying specific mechanisms that underlie the discordant phenotypes between human genetic disease and orthologous mouse mutant models may yield valuable insights into potential treatment strategies [40]. Although the physiological function of FPC remains undefined, we anticipate that continued study of *Pkhd1* mutant mouse models will expand our understanding of the mechanism(s) underlying mouse resistance to the severe renal disease that characterizes human ARPKD. Defining such mechanisms, in turn, could yield potential new targets for therapies that mitigate the full expression of this devastating human disease.

## Supplementary Information

Below is the link to the electronic supplementary material.

Supplementary file1 (DOCX 24 KB)

Supplementary file2 (PDF 4392 KB)

## Data Availability

The datasets generated during and/or analyzed during the current study are included in this manuscript and its supplementary information files.
