# Supplementary material for: Pkhd1cyli/cyli mice have altered renal Pkhd1 mRNA processing and hormonally sensitive liver disease
Source: J Mol Med (Berl). 2023 Aug 16;101(9):1141–51. doi: 10.1007/s00109-023-02351-2 (PMC10482757; doi:10.1007/s00109-023-02351-2)
Supplement: Supplementary file 1 — Supplementary file1 (DOCX 24 KB) [file 109_2023_2351_MOESM1_ESM.docx]

**Supplementary Table 1**

| **Primer Name** | **Primer Sequence (5’----3’)** |
| --- | --- |
| *Pkhd1-1F* | AACTTGCCCTGTGGTCATTT |
| *Pkhd1-1R* | CACTCCCACTTCCTTTTGCT |
| *Pkhd1-2F* | TGGGCTAGCACTACATTTAAATAATC |
| *Pkhd1-2R* | TGATGGCTTCACTCACATTCT |
| *Pkhd1-3F* | TCTGCTTTAAAGTATTGGATGTGC |
| *Pkhd1-3R* | TGTTCTTAAACAAAGAAGCGAGAA |
| *Pkhd1-4F* | CCTCAGGCAAACGCAATATC |
| *Pkhd1-4R* | TGCTAAGCAGAGCAAAGACTG |
| *Pkhd1-5F* | TCAAGTGGGAACTCCTGGTT |
| *Pkhd1-5R* | TGGGTTGACTCGTAACTTGC |
| *Pkhd1-6F* | CCAGTAATGCTTTGTGGGAAA |
| *Pkhd1-6R* | TGCTTACAGAAAACAATGGGATT |
| *Pkhd1-7F* | CTGTGCCTTGTTGGGGATAC |
| *Pkhd1-7R* | CCAGGATGCCATGTAGCAG |
| *Pkhd1-8F* | TGGGAACACCACACTATTGC |
| *Pkhd1-8R* | GGTTGTCCTATGGAAGGAACTG |
| *Pkhd1-9F* | GTTTCCTCTGGCCAATTTGA |
| *Pkhd1-9R* | TGGTTTTGTTTTCTTTTCTGGA |
| *Pkhd1-10F* | GGTAGCAATATCCTGGCTCA |
| *Pkhd1-10R* | TGAAGGAAAACCAGGAGGTG |
| *Pkhd1-11F* | GAACTCCCTTTTGAGAAGCTGA |
| *Pkhd1-11R* | TGCCAGAAGAAGAGGAGAGC |
| *Pkhd1-13F* | GGCATGGTGTGTTTGTGTGT |
| *Pkhd1-13R* | CCCTGGATCCTTGAGACAAA |
| *Pkhd1-14F* | GGGGCTTTGTTGTGAGGAG |
| *Pkhd1-14R* | AACAGTCAATCTGCACTTGCTA |
| *Pkhd1-17/18F* | ATGGAAACTGCCTGTTGAGG |
| *Pkhd1-17/18R* | CCATGCCTTTATTTGGCTCT |
| *Pkhd1-19F* | CTGAGGGACCAATGTCACTGT |
| *Pkhd1-19R* | GGTTTTGAGCCCAAGGAATA |
| *Pkhd1-20F* | ATGCAGCAAGCTCTCCTCAC |
| *Pkhd1-20R* | ATGAACCATTGTCCCCAAGA |
| *Pkhd1-21F* | CTCAGGCCTTGAGGAGGAG |
| *Pkhd1-21R* | GAAAGTCTGAGACAGGCATTTG |
| *Pkhd1-22F* | TGAGTGACAACCATTTCTAGCC |
| *Pkhd1-22R* | TCCCACCCTGATAGAAGCTG |
| *Pkhd1-23F* | GAAGAGCTGGATATGGTCTGC |
| *Pkhd1-23R* | CGCAGGCTATGTTTCCCTTA |
| *Pkhd1-24F* | TTTTCCTTTATCCTTGATGTGGA |
| *Pkhd1-24R* | CCAGTGACTGTTTTCACTCATACA |
| *Pkhd1-25F* | AAAAACTCATCTTTTGTTTTCATCA |
| *Pkhd1-25R* | AAGAGTCACAGGAGTTGAACCA |
| *Pkhd1-26F* | CAAATACTTTGAGTGCCCTTG |
| *Pkhd1-26R* | CATTCAGGCAAAACACTGGT |
| *Pkhd1-27F* | TGCAGTTGTCCGAGTGAGTC |
| *Pkhd1-27R* | GGCCTAGATTTCTGCCTATCAA |
| *Pkhd1-28F* | TTCCTGTAACTAACTTTTATCTGCCTA |
| *Pkhd1-28R* | CCAGAATTACAACTCCCAGCA |
| *Pkhd1-29F* | TTTCTTCTTTTGGCTGATTATGC |
| *Pkhd1-29R* | GAGCTCCAAACTAGCTGGACTAA |
| *Pkhd1-30F* | GAGAATTATTTTCAGGGCTGACTT |
| *Pkhd1-30R* | CAAAATAGAATTTCCCAGATGAGG |
| *Pkhd1-31F* | TCCTCCCTTTGCAAACATCT |
| *Pkhd1-31R* | AAGCCCTGACCTCACCTGAT |
| *Pkhd1-32-1F* | TGGGTCCTCAGGAATCAATC |
| *Pkhd1-32-1R* | AGTGACCACTGGTGTTGCTG |
| *Pkhd1-32-2F* | GGCTGGCAACACTTTCTTCT |
| *Pkhd1-32-2R* | CTGTCCCACCACATTTAGACC |
| *Pkhd1-32-3F* | TGGGGTTTGCCAATATGTCT |
| *Pkhd1-32-3R* | CCATGGTTAGAGACCCACTGA |
| *Pkhd1-32-4F* | CCAGCAAGTGTAAGGGGAAC |
| *Pkhd1-32-4R* | TCGTCAAGAGACTTCCACCA |
| *Pkhd1-32-5F* | TGCTTGCCTCAATAGTGTTTCT |
| *Pkhd1-32-5R* | CACGGTGATGTCCCAATAAA |
| *Pkhd1-32-6F* | TTCACCCCAGAATTGCTTTC |
| *Pkhd1-32-6R* | TGATGTTGGTACCTCTCTTCCT |
| *Pkhd1-33F* | GGCTGAATAGCAAGGGAATG |
| *Pkhd1-33R* | CAGAAATAGAAAGGACCAAAGGA |
| *Pkhd1-34F* | TCCTTGCTCAAAGAAAACATCA |
| *Pkhd1-34R* | GCTTTCTGACAGCTCTGTGG |
| *Pkhd1-35F* | AGCTAATGGCTCGCAATGAT |
| *Pkhd1-35R* | GCTTTGCTCCCTCAAAGATG |
| *Pkhd1-36F* | GCATTTAGCATTATCCATTGTCA |
| *Pkhd1-36R* | CTCTCCTTCCCATGCCTTG |
| *Pkhd1-37F* | CACACAAAAGCCATTTCATAACA |
| *Pkhd1-37R* | TGATTTACCAATGGTCTCTCTCTC |
| *Pkhd1-38F* | CTGTTCTTGGCTGGTGTTCC |
| *Pkhd1-38R* | TGCTAATGTATCGCATCAAGAGA |
| *Pkhd1-39F* | CTGCTTTGCATGGTGTGTCT |
| *Pkhd1-39R* | TGAAGAAGGTCAAACACAGTCC |
| *Pkhd1-40F* | GCAGTGAAACAAGATGGTTGG |
| *Pkhd1-40R* | TCCAGTCCCACATATCACCA |
| *Pkhd1-41F* | TTGTCAACAGATTCACAGGAGA |
| *Pkhd1-41R* | GGGGGATACTTTATGGGAGA |
| *Pkhd1-42F* | TGTACCCTTTCTCTGCCTCA |
| *Pkhd1-42R* | GGGCATGAATAGGCTTCAAA |
| *Pkhd1-43F* | CATTGAGCCATCTTCAGTGC |
| *Pkhd1-43R* | TGTTCCTGTGAACTGCTGGA |
| *Pkhd1-44F* | TCCACCATACATGAAAAGAATAGAA |
| *Pkhd1-44R* | TGTGCATAAAAGCCACAATCA |
| *Pkhd1-45F* | GCACCCCTTTAATTCCAATG |
| *Pkhd1-45R* | TGTTCTCTTCCTCATCAAGGTG |
| *Pkhd1-46F* | ATGCCAGTGTGCTCAATCAA |
| *Pkhd1-46R* | GGCCAGTATACATGATTAAGTGTTG |
| *Pkhd1-47F* | AGCAAGCGTTCTCTTGTCTTA |
| *Pkhd1-47R* | GACCCATAATACCTCTGCCACT |
| *Pkhd1-48F* | GAATCTTGTGGCTTTTAGTTGC |
| *Pkhd1-48R* | TCAGCCAGGGCTATACAGAGA |
| *Pkhd1-49F* | CCAGGTCCTATGGAAGAGCA |
| *Pkhd1-49R* | AAACGTTGCTGCAGTTTCCT |
| *Pkhd1-50F* | CCTGCTTCTCACACAGAACG |
| *Pkhd1-50R* | CAGCACAAATGCAGAAATTCA |
| *Pkhd1-51F* | TGAAGAATAAAACACAACACCATCA |
| *Pkhd1-51R* | AGCTGATGCTTGCCTGGTTA |
| *Pkhd1-52F* | AAAAAGAGAATCTTGAACTTGTCAC |
| *Pkhd1-52R* | TGCATTACATCATTCAATCTTATCC |
| *Pkhd1-54F* | CCATGCAGTCAGTATCATAGCA |
| *Pkhd1-54R* | TCTGTCTCTTTCTATTTGTCTCTCACA |
| *Pkhd1-55F* | CCTTAGATCTGGACAGACACCTT |
| *Pkhd1-55R* | GCATTTCATTTCACAATCCAA |
| *Pkhd1-56F* | TGCTGATGTGGGCTGTACTT |
| *Pkhd1-56R* | ATGAAACAGGCTGCTTATGG |
| *Pkhd1-57F* | TGTCACTTGTGAGGTACACTGG |
| *Pkhd1-57R* | GGTCCAGAATCACTTAGTAGCTG |
| *Pkhd1-58-1F* | TATTCCGATACCCCAAAGCA |
| *Pkhd1-58-1R* | TGCATAGTTCACTTTGATTCCTG |
| *Pkhd1-58-2F* | CGATGTGGAGGGTCAGAACT |
| *Pkhd1-58-2R* | TGTGATAGCCAAAAGACCAACA |
| *Pkhd1-58-3F* | TGCCATGGTTCAGACAGAGA |
| *Pkhd1-58-3R* | ACATTGTGTGTTTGGGTGGA |
| *Pkhd1-59F* | GGGTTGTCCTGGATAGCTCA |
| *Pkhd1-59R* | TGTGTTTGGGGATTTAAGCA |
| *Pkhd1-60F* | GCCCATGTGCTTTCTTCTT |
| *Pkhd1-60R* | GAACAACAGGGCCACCAG |
| *Pkhd1-61-1F* | GCATAACACATGGTGGTGAGA |
| *Pkhd1-61-1R* | GAAGAAAACGCAGGAATGGA |
| *Pkhd1-61-2F* | CTCCCACAAGCTCTCTGTC |
| *Pkhd1-61-2R* | CTCTCTCCCAGCCCTTTTCT |
| *Pkhd1-61-3F* | AGGAGAGGAGCCTGTTGAAA |
| *Pkhd1-61-3R* | TGAATGGGTTCACTGTTCCTT |
| *Pkhd1-61-4F* | CACCAGCAACAACTCTGGAA |
| *Pkhd1-61-4R* | TCTGGTGAATCCTTTGCAGTT |
| *Pkhd1-62F* | TGAGAAACAGCTACTGCAATGA |
| *Pkhd1-62R* | TGGCAGTCGCTCTGTAAATG |
| *Pkhd1-63F* | TTCAATAAGCCAAATTTGATGC |
| *Pkhd1-63R* | TTGCCAACTTCAGATGTTTTG |
| *Pkhd1-64F* | GATATTCGATTCCTTGTCTCCA |
| *Pkhd1-64R* | TGAAAGGACTTATTCGAATCACAA |
| *Pkhd1-65F* | TTTGCTTAGTAACTTCAAAAACAGC |
| *Pkhd1-65R* | GGGGGTATCATGGAGTTGTA |
| *Pkhd1-66F* | TGCCTTTACATTTAGGTCCTTG |
| *Pkhd1-66R* | ACAAAGAAGGGGCTGGAACT |
| *Pkhd1-67-1F* | GCTGTCATGATGGCCAAATA |
| *Pkhd1-67-1R* | CCTCTCTTGCCTGAGGTCTG |
| *Pkhd1-67-2F* | AACAGCAGTTGCTCAGATACCA |
| *Pkhd1-67-2R* | CCAAACAGATCAGACCTCTCCT |
