# Supplementary material for: Pkhd1cyli/cyli mice have altered renal Pkhd1 mRNA processing and hormonally sensitive liver disease
Source: J Mol Med (Berl). 2023 Aug 16;101(9):1141–51. doi: 10.1007/s00109-023-02351-2 (PMC10482757; doi:10.1007/s00109-023-02351-2)
Supplement: Supplementary file 2 — Supplementary file2 (PDF 4392 KB) [file 109_2023_2351_MOESM2_ESM.pdf]

Journal of Molecular Medicine

***Pkhd1**$^{cyli/cyli}$* **mice have altered renal *Pkhd1* mRNA processing and hormonally-sensitive liver disease.**

Chaozhe Yang[1], Naoe Harafuji[1], Ljubica Caldovic[2,8], Weiying Yu[1], Ravindra Boddu[3,4], Surajit Bhattacharya[2], Hayk Barseghyan[2,8], Heather Gordish-Dressman[1], Oded Foreman[5,6], Zsuzsa Bebok[6], Eva M. Eicher[7], and Lisa M. Guay-Woodford[1,2*]

***Corresponding author:**

Lisa M. Guay-Woodford, M.D.

Current address: Children's Hospital of Philadelphia

guaywoodfl@chop.edu

Communicating author:

Naoe Harafuji, PhD

NHarafuji@childrensnational.org

Fig. 1

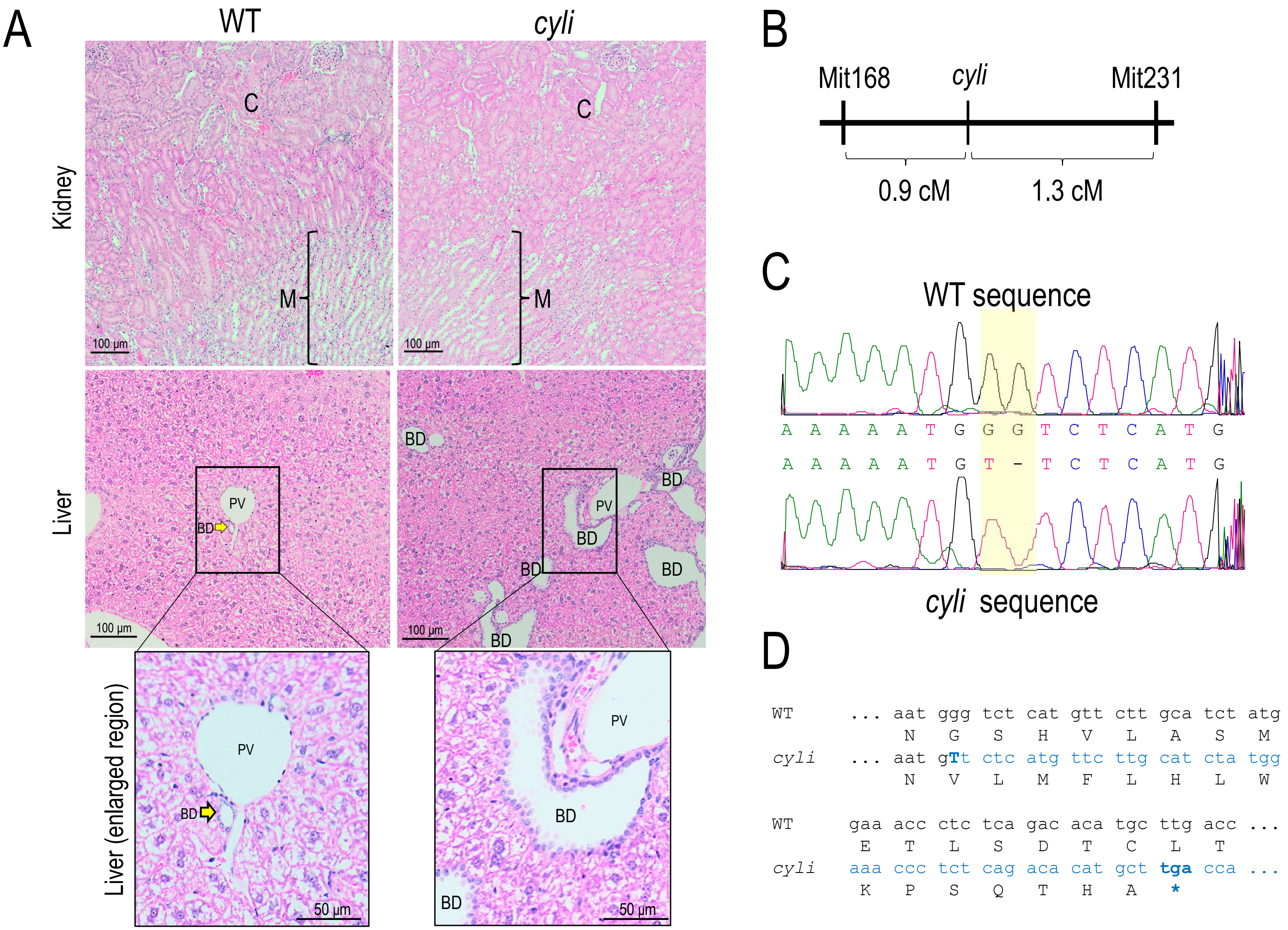
A
WT
cyli
Kidney
C
M
100 µm
Liver
PV
BD
100 µm
Liver (enlarged region)
50 µm
B
Mit168
cyli
Mit231
0.9 cM
1.3 cM
C
WT sequence
A A A A A T G G G T C T C A T G
A A A A A T G T - T C T C A T G
cyli sequence
D
WT ... aat ggg tct cat gtt ctt gca tct atg
N G S H V L A S M
cyli ... aat gTt ctc atg ttc ttg cat cta tgg
N V L M F L H L W
WT gaa acc ctc tca gac aca tgc ttg acc ...
E T L S D T C L T
cyli aaa ccc tct cag aca cat gct tga cca ...
K P S Q T H A *

Fig. 2

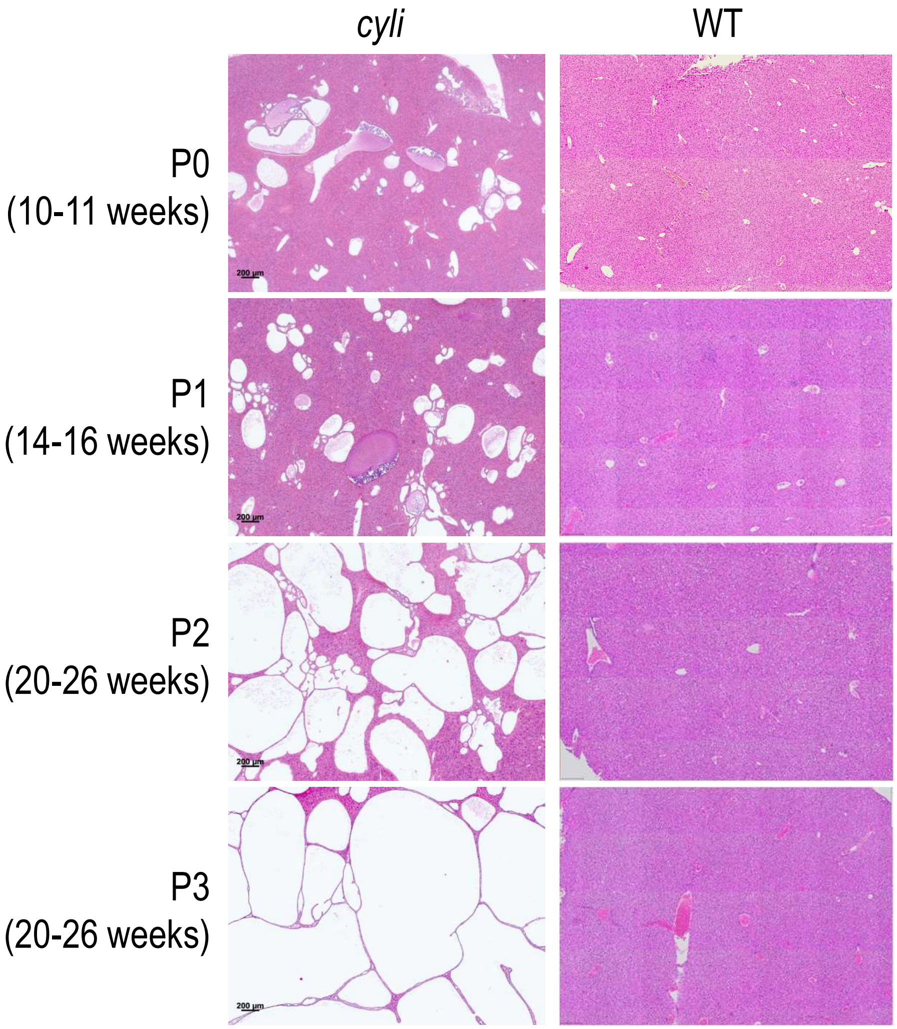

cyli
WT
P0
(10-11 weeks)
P1
(14-16 weeks)
P2
(20-26 weeks)
P3
(20-26 weeks)

Fig. 3

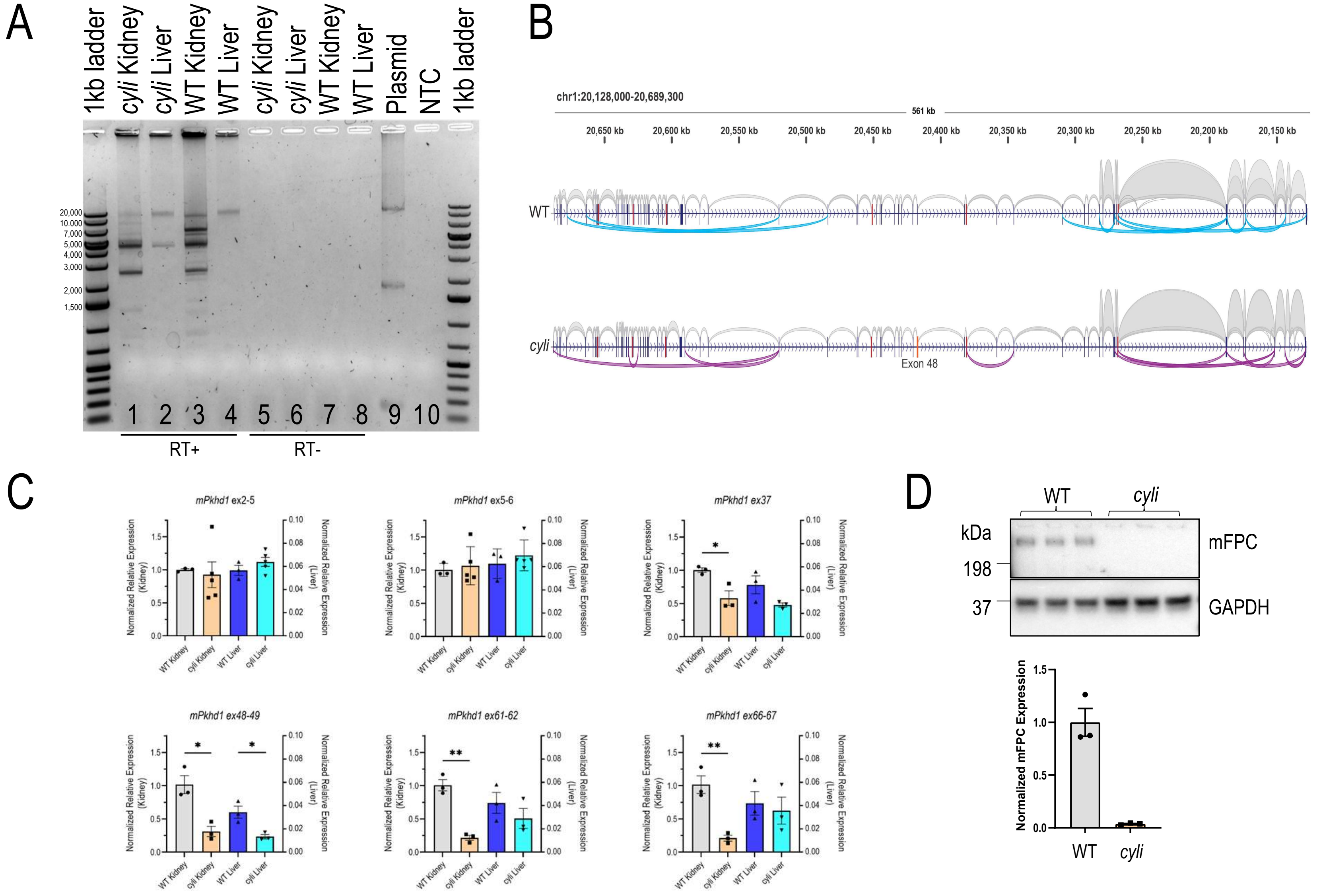

A
1kb ladder
cyli Kidney
cyli Liver
WT Kidney
WT Liver
cyli Kidney
cyli Liver
WT Kidney
WT Liver
Plasmid
NTC
1kb ladder
20,000
10,000
7,000
5,000
4,000
3,000
2,000
1,500
1 2 3 4 5 6 7 8 9 10
RT+
RT-
B
chr1:20,128,000-20,689,300
561 kb
20,650 kb
20,600 kb
20,550 kb
20,500 kb
20,450 kb
20,400 kb
20,350 kb
20,300 kb
20,250 kb
20,200 kb
20,150 kb
WT
cyli
Exon 48
C
mPkhd1 ex2-5
mPkhd1 ex5-6
mPkhd1 ex37
mPkhd1 ex48-49
mPkhd1 ex61-62
mPkhd1 ex66-67
Normalized Relative Expression (Kidney)
Normalized Relative Expression (Liver)
WT Kidney
cyli Kidney
WT Liver
cyli Liver
D
WT
cyli
kDa
198
37
mFPC
GAPDH
Normalized mFPC Expression
WT
cyli

Supplementary Fig. S1

Supplementary Fig. S1 ONT sequence distribution. Length distribution of *Pkhd1* cDNA sequenced using Oxford Nanopore Technology (ONT). The PCR amplicons from the kidneys of 4.5-week-old WT and *cyli* female mice were sequenced using the ONT long-read sequencing method. The X-axis shows the read length bins and the Y-axis shows the number of kilobases (kb) in each bin. Distribution of read lengths from sequencing WT *Pkhd1* transcripts showed maxima at approximately 2, 4, 6, and 11 kb (upper panel), which was consistent with WT *Pkhd1* cDNA amplification products (**Fig. 3A**). Distribution of read lengths from sequencing *cyli Pkhd1* transcripts had maxima at approximately 2 and 4.5 kb, and the full length 12 kb *Pkhd1* transcripts (lower panel). The results were graphed using GraphPad Prism version 9.5.1 for Mac.

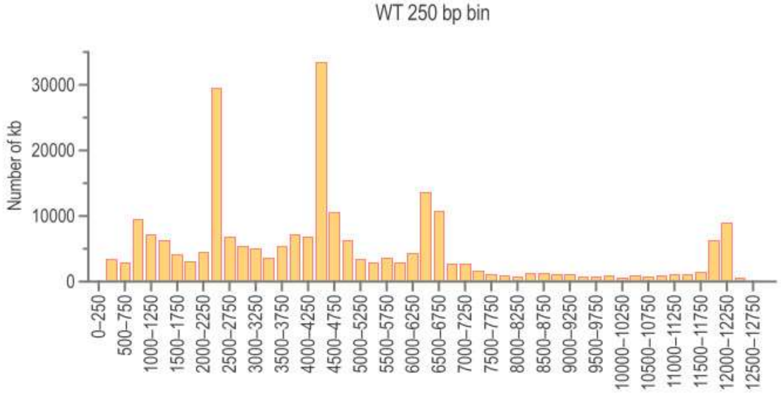

Reads: 131.98 k
Total DNA: 298.03 Mb

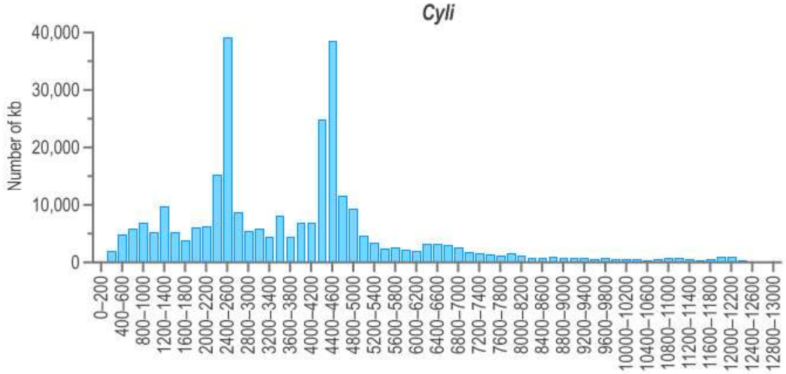

Reads: 181.58 k
Total DNA: 440.23 Mb

Supplementary Fig. S2

Supplementary Fig. S2 FPC abundance is below detection limit in WT and *cyli* livers. Immunoblot of FPC protein in liver lysates from 2-week-old WT and *cyli* male mice using a rat monoclonal primary antibody specific for the FPC carboxyterminus. FPC specific bands were not observed in either WT or *cyli* liver lysates likely due to a small fraction of cholangiocytes in the liver tissue. The lower molecular weight bands observed in the FPC immunoblot were likely non-specific cross-reacting proteins. Actin was used as a loading control. n=2 mice per group.

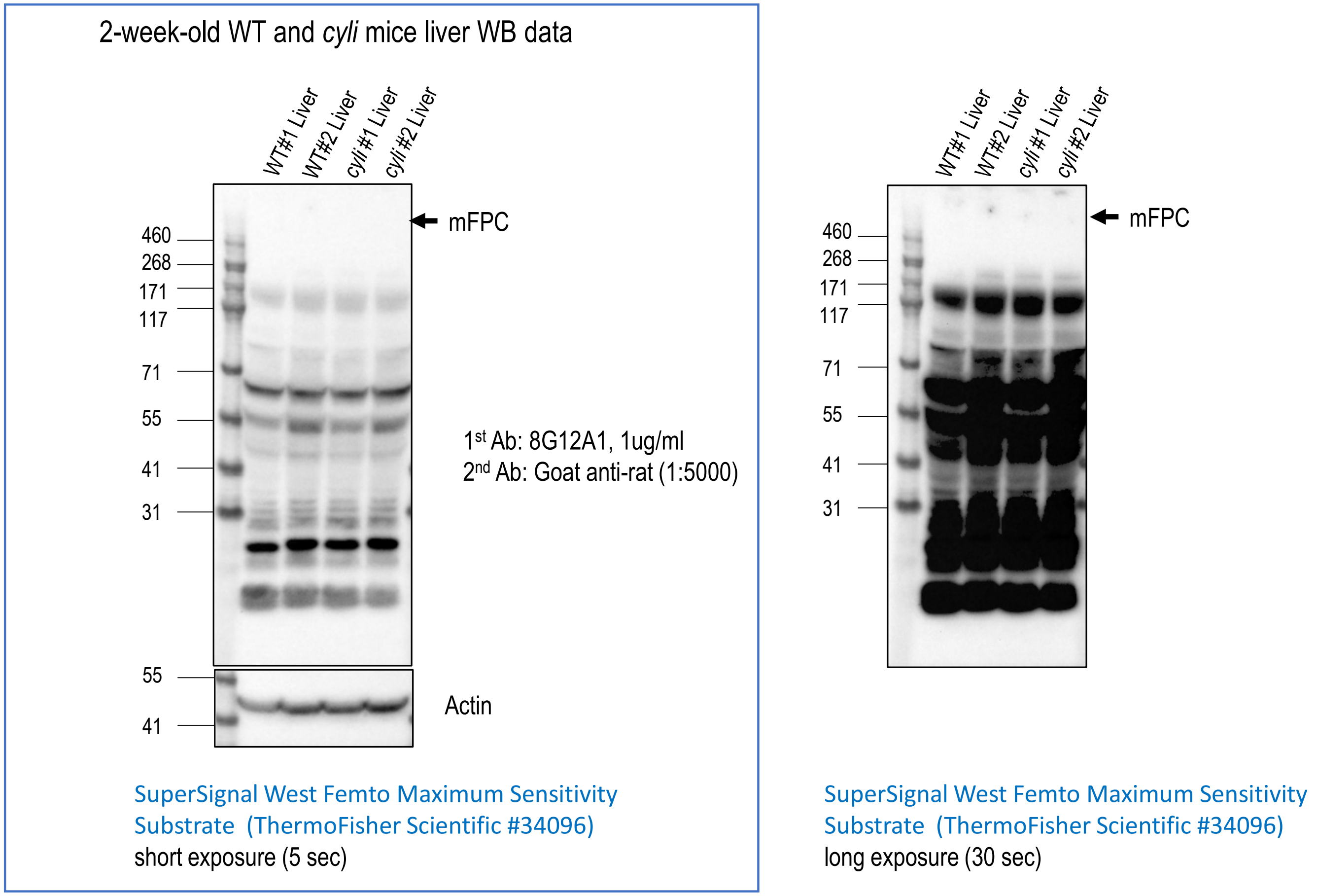

Supplementary Fig. S3

Supplementary Fig. S3 ***Pkhd1*** **transcript structure.** Schematic representation of the exons in the longest ORF encoding major functional protein coding domains. The ORF is preserved when splicing occurs between exons with similar configurations (like-to-like colored/shaped exon boundaries), e.g., exon 6 to 7; exon 6 to 48, which could generate both the longest ORF transcript and alternatively spliced transcripts. The *cyli* mutation and resulting downstream PTC are indicated by the red **X**.

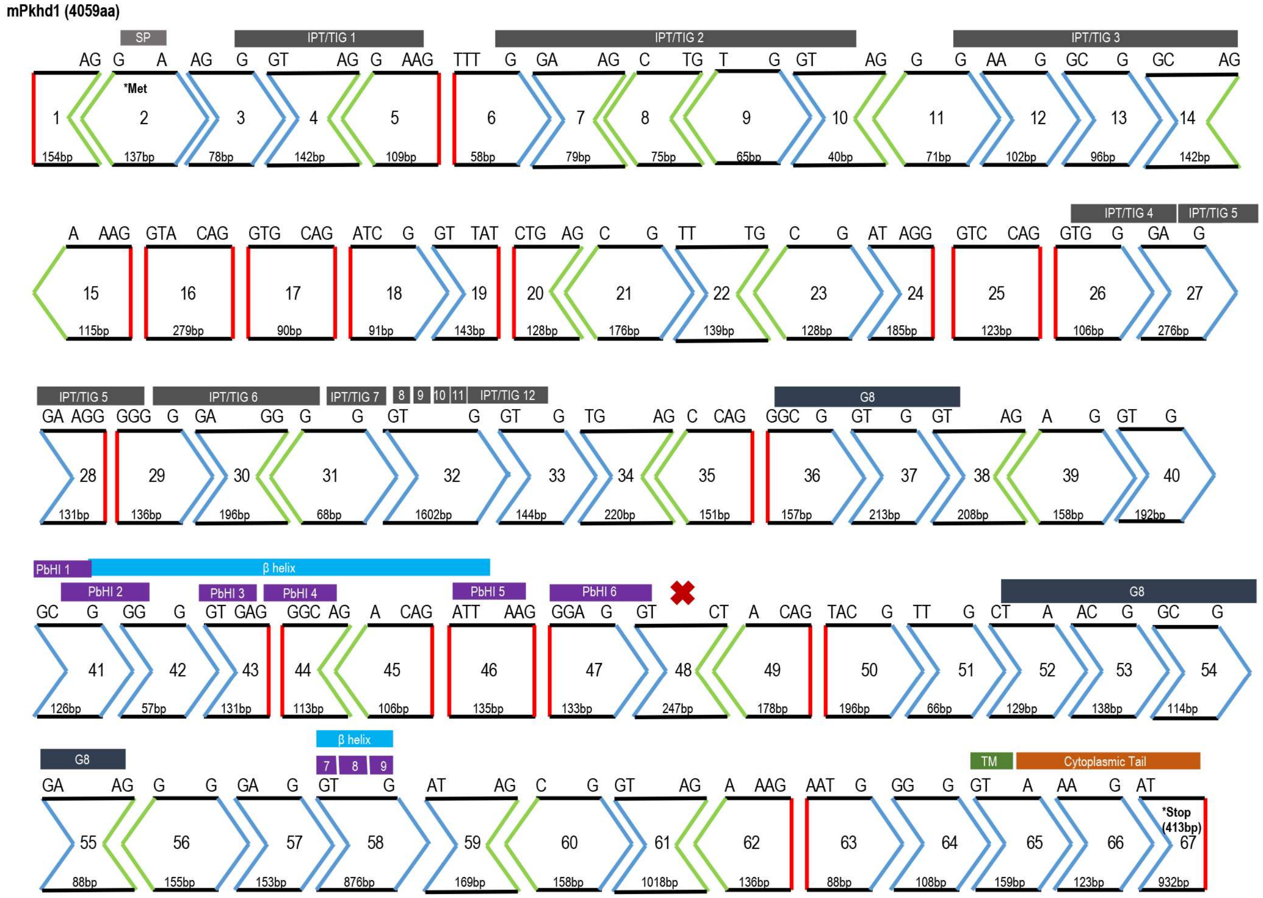

Full unedited membranes for Fig. 3D

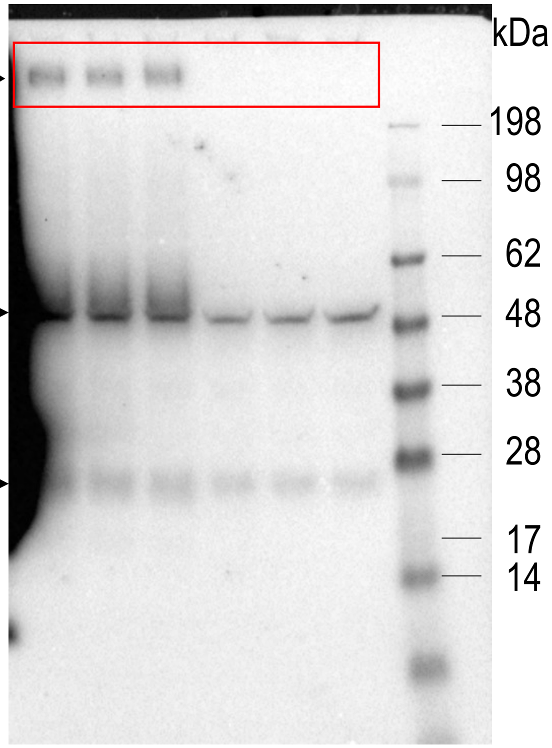

NOTE: Low molecular weight bands (arrowheads) correspond to IgH and IgL chains detected by anti-mouse secondary antibody reagent.

NOTE 2: SuperSignal West Dura Extended Duration Substrate (ThermoFisher Scientific # 34076).

Exposure time is 150 sec.

1st Ab: 8G12A1, 0.5ug/ml

2nd Ab: Rat secondary(1:5000)

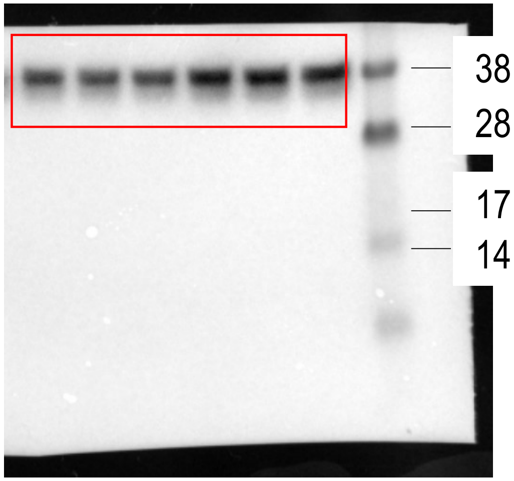

Full unedited membranes for Fig. S2

FPC, short exposure (5 sec)

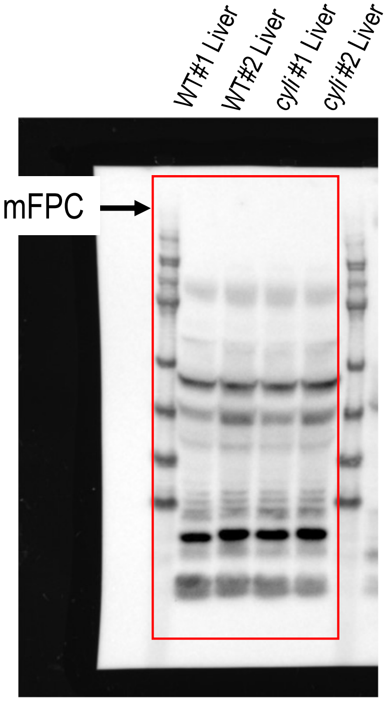

FPC, long exposure (30 sec)

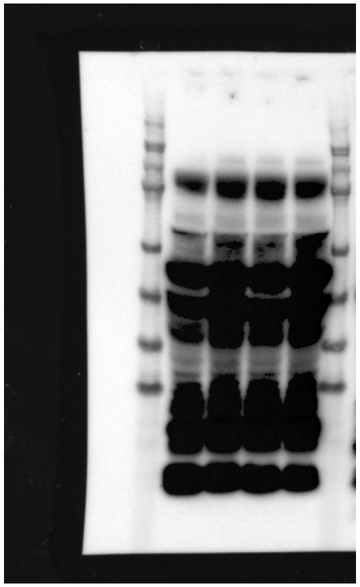

Actin

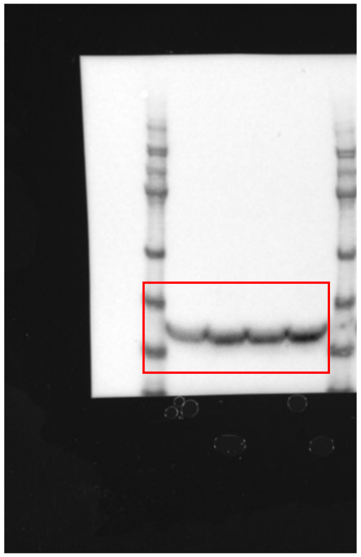

FPC Images developed with SuperSignal West Femto Maximum Sensitivity Substrate (ThermoFisher Scientific #34096)
